# Management of Serious Adverse Events Following Deoxycholic Acid Injection for Submental and Jowl Fat Reduction: A Systematic Review and Management Recommendations

**DOI:** 10.1093/asjof/ojae061

**Published:** 2024-08-13

**Authors:** Sachin M Shridharani, MacKenzie L Kennedy

## Abstract

Pivotal Phase 3 randomized control trials have demonstrated a favorable safety profile for ATX-101 in submental fat (SMF) reduction; however, in real-world settings, several serious adverse events (SAEs) have been reported, most of which are procedure related and avoidable. Current understanding of the management of uncommon AEs and SAEs is based on the aesthetic surgeon's discretion, and overzealous protocols for sclerosis agents are being applied for ATX-101-induced arterial injury. This review focuses on showcasing the management of SAEs reported previously and updating it with personal clinical experiences with ATX-101 for SMF and jowl fat reduction. Along with adherence to the standard procedures for ATX-101 administration, the authors recommend investigating other potential causes of SMF accumulation and jowling mechanism, appropriate demarcation of the surface area to determine the number of vials, and assessment of the fat pad thickness to determine the number of required treatment cycles for optimal therapeutic outcomes. Surgery is preferable for jowling caused by compartment displacement (ptosis), whereas fat-reducing treatments such as ATX-101 are contraindicated for jowling caused by subcutaneous tissue atrophy. Some proactive measures that can be employed to prevent AEs include avoiding intradermal injections to prevent skin ulceration/necrosis, injecting lidocaine to check for smile asymmetry as an indication of marginal mandibular nerve proximity, administering 1 to 2 mm deeper injections in males to prevent alopecia, employing good aseptic techniques to prevent abscess formation, injecting 1 product at a time using correctly labeled syringes, and confirming the diagnosis of pyoderma gangrenosum before treating it as an infection.

**Level of Evidence: 3:**

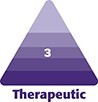

Deoxycholic acid (DCA; ATX-101; Kybella; Allergan, Inc., Irvine, CA), a nonspecific cytolytic agent, was the first injectable therapy approved in 2015 for improving the appearance of moderate-to-severe convexity/fullness associated with submental fat (SMF) in adults.^1^ Considering that the chin and jawline are common areas of aesthetic concern, jowl fat is also being targeted (off-label) as an important component of facial rejuvenation.^2-4^

When injected subcutaneously, ATX-101 promotes adipocytolysis, owing to its accentuated affinity for adipose tissue membranes over other tissues.^5,6^ Consequently, this adipocytolysis-induced local inflammatory response leads to soft-tissue tightening through the removal of cell debris and deposition of collagen following the recruitment of fibroblasts to the injection site.^7^

Randomized controlled trials (RCTs)^8-13^ of DCA injections in the SMF area have reported adverse events (AEs) that were mostly transient, mild, or moderate in severity and localized to the injection site.^1^ The management paradigms targeting common unavoidable injection-site AEs with ATX-101 have been detailed by Dover et al^14^ and Fagien et al.^15^

However, since the approval of ATX-101 (2015), avoidable, uncommon, and serious AEs (SAEs) of ATX-101 have been reported in the real-world setting. These AEs were mostly procedure-related consequences of direct cytolysis. The corresponding author has performed ∼5000 procedures with ATX-101, with publications focusing on SMF, jowl fat, and body/extremity fat reduction. In addition, over the past 2 years, many cases presenting uncommon AEs and SAEs have been referred to the corresponding author's clinic, making it a center of excellence in resolving ATX-101-associated SAEs. Therefore, in this review article, the authors discuss and highlight the best practices for the prevention of and management strategies for resolving SAEs associated with ATX-101 use in SMF and jowl fat reduction.

## METHODS

### Search Strategy and Selection Criteria

The authors conducted a systematic literature search for studies reporting SAEs associated with ATX-101 use for SMF/jowl fat reduction. All studies published before July 26, 2023, and indexed in the MEDLINE/PubMed database were evaluated. The search string for MEDLINE/PubMed was as follows: (adverse events) AND (ATX-101 OR deoxycholic acid OR KYBELLA OR BELKYRA).

The US Food and Drug Administration prescribing information lists the most common AEs expected with ATX-101^1^; however, for this review, the authors are considering uncommon AEs as SAEs and discussing their treatment algorithms in comparison with what has already been published in the literature. The definition of SAEs is in accordance with the International Council for Harmonisation E6 (R1) Guideline for Good Clinical Practice.^16^

## RESULTS

A total of 281 records were identified for screening, of which 26 were included for data extraction.

### Anatomy

#### Submental Fat

SMF is located in a distinct compartment within the preplatysmal fat and is demarcated by the submental crease, caudal continuation of labiomandibular fold, and cervicomandibular angle.^17,18^

#### Jowl

Jowl fat consists of the superior and inferior fat pads: the superior fat pad extends from the nasolabial fold and approximates the oral commissure, whereas the inferior fat pad abuts the superior fat pad and reaches the mandibular border inferiorly.^19^ The mandibular septum acts like a sling beneath both jowl fat pads, separating them from the submandibular fat pad.^19^ The inferior jowl fat pad overlays important neurovascular structures at the mandible, such as the facial artery and vein (which cross the mandible at the antegonial notch)^20^ and the marginal mandibular nerve (MMN) that runs beneath the platysma muscle along the mandible superficial to the facial artery and vein.^21^ However, cadaver studies show that in 52% of cases, the MMN follows the angle of the mandible; in 32% of cases, it is found ∼1.1 to 1.6 cm below the mandible; in 16% of cases, it runs above the mandible; and when anterior to the facial artery, it is always above the inferior border of the mandible.^21,22^

### Technique Options

Treatment options for unwanted fat deposits resistant to diet and exercise and applicable to SMF/jowls include invasive treatment procedures such as surgery, laser lipolysis, fat excision, or liposuction, and noninvasive treatments such as focused high-frequency ultrasound, radiofrequency, cryolipolysis, or localized injections of lipolytic drugs.^23-28^

### Evaluations

#### Preprocedural Assessment and Precautions Recommended to Avoid SAEs

As described by Teller et al, a general examination before ATX-101 administration should include assessing the patient's SMF position in the Frankfort plane; evaluating platysma and digastric muscle contraction by making the patient say “E”; palpating the SMF for masses, irregularities, and moderate-to-severe submandibular glands; examining preplatysmal fat for better outcomes; assessing asymmetry during smiling; evaluating severe submental skin laxity; identifying long/wide platysma bands; examining the lateral vs lower position of the hyoid bone; checking for digastric muscle hypertrophy by making the patient place their tongue on the roof of their mouth; and checking for mandibular hypoplasia.^29^

In addition, it is advisable to exclude patients with other potential causes of submental convexity/fullness (eg, thyromegaly, cervical lymphadenopathy, submandibular gland ptosis, and excessive skin laxity), those with prior use of injectable lipolytic agents, those using anticoagulants, or pregnant patients. If changes in anatomy, landmarks, or scar tissue are observed, caution should be exercised as they may directly impact the desired aesthetic results or the ability to safely administer DCA injections. Of note, the only contraindication of ATX-101 is the presence of infection at the injection sites.^1^ Facial markings used to isolate the jowl have been published previously.^4^

It is important to address the jowling mechanism through visual inspection and palpation of the area for each patient: surgery is preferable for jowling caused by compartment displacement (ptosis), whereas fat-reducing treatments such as ATX-101 are contraindicated for jowling caused by subcutaneous tissue atrophy.^30^ However, jowling caused by fat flow across the mandible,^30^ which is perceived as both visible focal fullness and palpable as discernible subcutaneous fat, is ideal for fat reduction with ATX-101. Other aspects that need to be considered are patient preference for a particular treatment modality, patient expectations, and multiple age-related changes requiring surgical rejuvenation.^4^

In addition, patients with submental fullness caused by skin laxity and platysma diastasis should not be treated with ATX-101. Similarly, patients with submental fullness caused by prominent submandibular glands, postplatysmal fat, or digastric muscle hypertrophy do not show complete improvement with ATX-101. However, ATX-101 can be used as an adjunctive treatment in these cases after surgical rejuvenation of the aging face to reduce residual fat along the jawline and jowls.^2^

**Figure 1.**
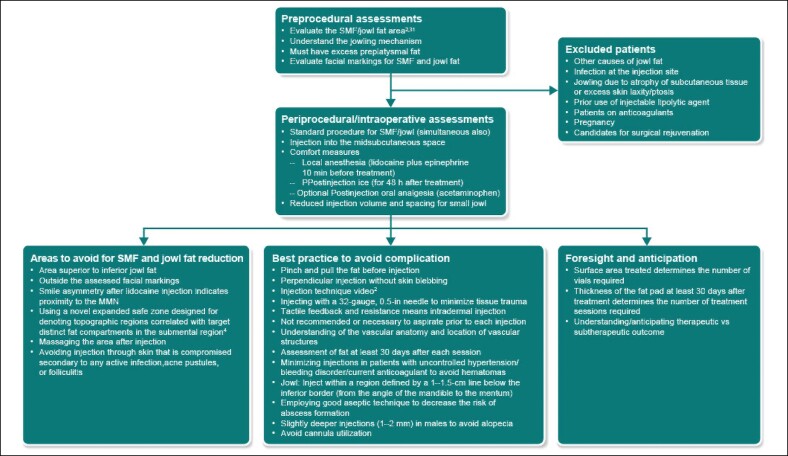
Best practice to manage unavoidable AEs and prevent avoidable SAEs. AE, adverse event; MMN, marginal mandibular nerve; SAE, serious adverse event; SMF, submental fat.

Overall, given that multiple treatment sessions will be required, it is advisable to anticipate the number of sessions required. Based on results from RCTs,^8-13^ patients may require a maximum of 6 treatment sessions at least 1 month apart for SMF reduction. The surface area treated indicates the number of vials required to achieve the desired therapeutic outcome. Per protocol, if the needle penetrates the skin, 0.2 mL of ATX-101 is used per injection site. The number of treatment cycles is determined by the thickness of the fat pad. Therefore, it is important to understand whether the treatment is therapeutic/subtherapeutic, because a subtherapeutic treatment can result in suboptimal outcomes (Figure 1).

#### Periprocedural/Intraoperative Assessments and Strategies to Avoid SAEs

Standard procedural details for SMF and novel approaches to demarcate and target jowl fat have been published in the literature.^1-3^ Of note, ATX-101 treatment for jowl fat and SMF reduction can be initiated simultaneously.^2^ When targeting the jowl, it is advisable to pinch and pull the fat and skin away from the underlying musculature and neurovascular structures during injection. This allows for perpendicular injection into the jowl fat midway into the subcutaneous fat (∼6-10 mm), thereby optimizing the angle of injection, and decreases the likelihood of temporary MMN paresis.^2^

An appropriate injection technique is critical for successful jowl treatment and has been described previously using an online video.^2,4^ Of note, dermal blebbing is an indicator that ATX-100 injections are too superficial and requires that the needle be gently pushed deeper into the underlying fat. It is advisable to not be overly aggressive and reduce both the injection volume and spacing for the treatment of small jowl surface areas and to not target the region superior to the isolated marking of the jowl fat pad. In addition, massaging the area after injection is not recommended as it may displace ATX-101 into surrounding nontarget tissues.^4^

The corresponding author believes that comfort measures are important to reduce AEs; the use of local anesthesia (lidocaine plus epinephrine 10 min before treatment), postinjection ice (for 48 h after treatment), and postinjection oral analgesia (acetaminophen) is likely to reduce the intensity of all unavoidable AEs, such as pain, edema, and bruising.^2^

In addition to MMN injury, an asymmetric smile may also be caused by damage to the cervical nerve branches; this occurs when ATX-101 is injected too deeply to reach the platysmal surface, leading to muscle injury and dysfunction.^19^ Therefore, it is recommended that ATX-101 injections target the midsubcutaneous space. To determine the need for additional treatment sessions, patients should be assessed at least 30 days after each treatment session.^2^

Intradermal injections can be prevented by appreciating tactile feedback and resistance during the injection. These injections have a higher resistance than subcutaneous fat, which has a lower tissue density and easier push pressure. Additionally, intradermal injections have a visible intradermal wheel, which is an appropriate visual cue for treatment.

Hematomas can be prevented by minimizing injections in patients with uncontrolled hypertension and those who are currently anticoagulated or have a history of documented bleeding. Pretreatment of the target tissue with lidocaine and epinephrine minimizes discomfort, which often leads to clenching and hypertension, along with the vasoconstrictive benefit of epinephrine. Additionally, using a 32-bore 0.5 in needle for injection minimizes tissue trauma (Figure 1).

### Complications and Safety Issues

#### Management of SAEs

**Figure 2.**
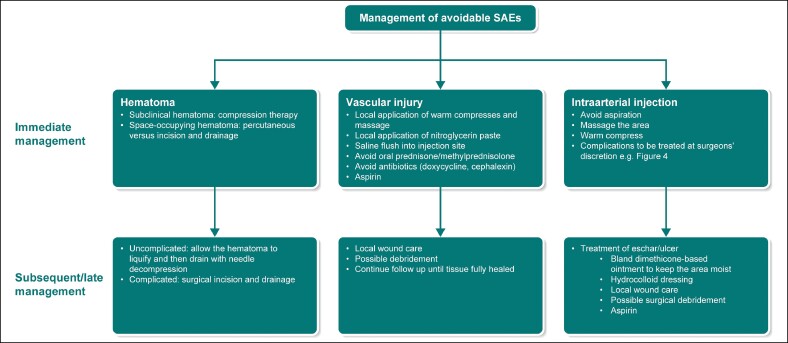
The management of avoidable SAE. SAE, serious adverse event.

*Hematoma*: Unfortunately, clinical trials of ATX-101 have failed to differentiate between hematoma and bruising, which are reported in 72% of patients treated with ATX-101 for SMF reduction.^1^ The prescribing information advises caution regarding the use of ATX-101 in patients with bleeding abnormalities or those who are currently being treated with antiplatelet/anticoagulant therapy.^1^ However, it is advisable to delay/avoid the procedure until laboratory/platelet findings normalize. The treatment of bruising has been published in the literature.^14,15^ Immediate treatment approaches for hematoma include compression therapy for subclinical hematomas, whereas space-occupying hematoma may require percutaneous vs incision and drainage. Thereafter, if the overlying skin and/or underlying critical structure does not comprise structures such as the neurovascular bundle/airway/functional joint, the hematoma can be allowed to liquify and then drained with needle decompression. Alternatively, surgical incision and drainage may be indicated (Figure 2).

*MMN injury*: Pivotal clinical trials and case reports of ATX-101 for SMF reduction have reported MMN injury, presenting as an asymmetric smile/facial muscle weakness (paresis), which resolves spontaneously without sequelae.^10,13,31^ In clinical trials, the incidence of MMN paresis was 4% (20/513) in the ATX-101 group and <1% (1/506) in the placebo group, with resolution occurring within a range of 1 to 298 days (median: 44).^1^ One severe case of MMN paresis that occurred in a patient treated with ATX-101 resolved within 85 days; this patient continued to receive additional treatment and was satisfied with the therapeutic outcome. The median duration of mild MMN paresis was 41.0 days (range, 7.0-60.0 days). For moderate MMN paresis events in 3 patients, the median duration of recovery was 56.5 days (range, 52.0-61.0 days) in 2 patients and 115.0 days in 1 patient.^13^

Similarly, a pooled analysis of 2 Phase 3, double-blind, RCTs for SMF reduction reported injection-site nerve injury in 2.1% (5/243) of cases in the high-dose (2 mg/cm^2^) group vs none in the low-dose (1 mg/cm^2^) group. Among them, 1 case was deemed as treatment-related SAE and presented as a temporary asymmetric smile on the right side of the face, possibly associated with injury to the MMN branch. This case did not require hospitalization or further treatment and resolved without sequelae.^11^ A similar rate (2%; 2/100) of MMN paresis was reported in a prospective observational study in patients from private practice to assess real-world early experience with initial treatment sessions of DCA injections for SMF reduction; both patients were from the single-treatment session group and fully recovered after 17 and 22 days.^32^

When using a novel expanded safe zone designed for denoting topographic regions correlated with target distinct fat compartments in the submental region, only 4.8% (8/167) of patients experienced MMN paresis during the first treatment session. The MMN paresis resolved with a median duration of 27 days (range, 14-40 days). The rate of MMN paresis differed by zones, with 62.5% (5/8) occurring in Zones S1 and S2 and 37.5% (3/8) occurring in Zones S1 to S3.^33^

When targeting jowls (*n* = 135), the author observed MMN paresis in 3.7% of the sites (*n* = 10), which lasted for a mean (standard deviation [SD]) duration per session of 8.5 (8.4) days. The mean (SD) volume of ATX-101 injected in a single session was 1.8 (0.6) mL (*n* = 60) and that in multiple sessions was 1.6 (0.7) mL (*n* = 210). A similar incidence rate was reported among 66 patients (incidence number [%]: 3 [5%]) in another study targeting jowl fat; the MMN paresis resolved in all 3 patients within a mean period of 26 days (range, 14-40 days).^2^

**Figure 3.**
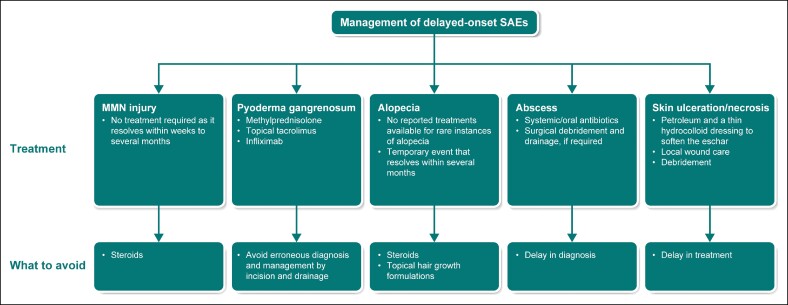
Management of delayed-onset SAEs. MMN, marginal mandibular nerve; SAE, serious adverse event.

To avoid injury to the MMN branch of the facial nerve, it is advisable to inject within a region defined by a 1 to 1.5 cm line below the inferior border (from the angle of the mandible to the mentum). The corresponding author recommends proper isolation of the jowl fat and use of appropriate injection techniques to avoid injury to the MMN, which is located superficial to the facial artery and vein and deep underneath the subcutaneous fat and platysma muscle. The pinch and pull technique can isolate the target tissue from underlying neurovascular structures; moreover, injecting a small volume of lidocaine with epinephrine before ATX-101 administration can help identify whether the injection site is close to the MMN. Smile asymmetry observed after lidocaine injection indicates that the depth of the ATX-101 injection should be adjusted superficially to avoid this area (Figure 3).^4^

*Alopecia*: During the corresponding author's early experience with initial treatment sessions of DCA/ATX-101 injections for SMF reduction, transient alopecia at the injection site was noted in 8 of 39 male patients (multiple treatment sessions: 23.8% [5/21]; single-treatment sessions: 16.7% [3/18]; *P* = .702).^34^ One additional case with transient alopecia at the injection site lasting for 6 weeks to 12 months was noted during the 2-year follow-up of the same set of patients (9/39 male patients; single sessions: 1/11 [9.1%]; multiple sessions: 8/28 [28.6%]; *P* = .136). In these cases, alopecia was transient (6 weeks-12 months) and did not require treatment.^32^ However, 1 case report does mention persistent DCA-induced patchy alopecia in the submental region that was refractory to treatment with topical bimatoprost after 11 months.^35^ The only procedural difference between that case report and the corresponding author's practice was the use of a 30 G needle vs a 32 G needle.^35^

When using a novel expanded safe zone to target distinct fat compartments in the submental region, 4.2% (7/167) of patients experienced alopecia, which resolved within a median duration of 143 days (range, 55-204 days).^33^ Thereafter, a couple of case reports have been published: 1 reported new-onset, transient, nonscarring submental alopecia occurring 1 week after the procedure, which did not affect subsequent treatment sessions and resolved ∼7 months after the first treatment session, and new-onset, nonscarring submental alopecia occurring 3 weeks after the procedure, with 60% improvement in alopecia after 1 year and 2 months from the first treatment session^36^; and other reported hair loss after 2 weeks of the procedure despite performing a punch biopsy of the lesion, although the outcome was not reported.^37^

In the corresponding author's experience, the outcome of alopecia while targeting jowls with ATX-101 was similar to that of SMF, as 3 out of 66 patients (5%) experienced transient injection-site alopecia that resolved within a mean period of 95 days (range, 64-126 days).^2^

Currently, there are no reported treatments available for rare instances of alopecia; however, to date, all cases have been temporary and take several months to resolve. To prevent alopecia, a slightly deeper injection of ∼1 to 2 mm should be employed in hair-bearing segments of the skin, as the male dermis tends to be thicker than the female dermis, with the hair bulb deeply rooted in the reticular layer of the dermis (Figure 3).

*Vascular injury*: To date, 3 cases of vascular injury associated with ATX-101 use for SMF reduction have been reported. The first case was of a female in her 20 s who underwent jowl and SMF reduction treatment in the same treatment session. Although the injections targeting the jowl were uneventful, the patient experienced immediate pain after a midline submental injection. On examination, blanching was observed extending from the neck to the lower mucosal lip, followed by dusky retiform discoloration noted within a few minutes. Immediate treatment comprised local application of warm compresses and massage. Although the discoloration improved over the next 30 min, swelling in the affected area was managed with oral prednisone administered the following day, leading to complete resolution after several days.^38^

In the second case, a female patient in her 40 s complained of left lateral neck pain at the injection site that radiated to the lower lip and teeth toward the end of her second DCA treatment session for SMF reduction. Immediate evaluation revealed dusky discoloration around the injection site. The patient was treated with local application of nitroglycerin paste and warm compresses, which improved the symptoms. In addition, the patient was treated with oral methylprednisolone, doxycycline, and aspirin on the same day. After 3 days, the patient returned to the clinic with an asymmetric smile, residual retiform purpura, and pinpoint hemorrhagic vesicles in the area. The patient was treated with local application of fluocinolone ointment and emollient twice daily. Complete resolution of the MMN paresis and purpura was observed after 2 weeks, with no permanent sequelae.^38^

In the third case, a 37-year-old female with a history of prior DCA injections into the submental region complained of sudden pain after a DCA injection during her first treatment session for moderate SMF reduction. Approximately, one-eighth of 0.2 mL of DCA was injected, after which a white linear blanching of the skin was observed running laterally and upward from the injection site. Immediate treatment comprised massaging the blanched area following needle removal; after a few minutes of massaging the affected area, a 2 cm erythematous patch was observed lateral and inferior to the injection site. Although the patient was comfortable, the white linear blanched area persisted after 10 min, for which a 2% nitroglycerin paste was applied. The following day, pictures sent by the patient showed that the previously white blanched area and medial injection site ecchymosis had now become a flat erythematous patch. Although there was slight paresthesia of the submental chin, it did not extend laterally to the erythematous patch. Subsequently, aspirin was initiated, along with warm compresses and oral corticosteroids, as the sequence of events resembled that of a vascular occlusive event. The lesion was more erythematous, with a papular eruption on Day 5. To avoid concerns related to the development of blisters and necrosis, the patient was initiated on a 10 day course of cephalexin. After 4 weeks, complete resolution of erythema was observed, which did not require laser treatment.^39^

These cases indicate that patients experiencing vascular injury without necrosis/ulceration can make a full recovery (Figure 2).

*Skin ulceration/necrosis*: The Phase 3 RCT, REFINE-2, reported 1 AE of mild superficial skin ulceration in a patient treated with ATX-101 for SMF reduction during the sixth treatment session, which resolved within 23 days.^13^

Subsequently, 3 case reports of skin ulceration/necrosis associated with DCA injections for SMF reduction have been reported. The first case was that of a 45-year-old female presenting with a wound on the central anterior neck after her second treatment session with DCA for SMF reduction that was performed at an outpatient plastic surgery clinic. Immediately after the treatment session, the patient complained of severe pain and discomfort. A few days later, the patient reported increasing redness and induration, followed by the development of a wound and eschar at the treatment site. On examination, a 2 cm violaceous nodule with central eschar was observed on the anterior neck, surrounded by moderate induration. Initial treatment involved the application of petrolatum and a thin hydrocolloid dressing to soften the eschar. The eschar fell off after 3 weeks, revealing a hypertrophic pink scar, which was treated with topical steroids for 2 months, intralesional triamcinolone injection (0.2 mL [20 mg/mL]), and pulsed dye laser treatment. Although this treatment approach resulted in significant aesthetic improvement, the management of the hypertrophic scar was continued.^40^

The second case was that of a 30-year-old female who presented with multiple depressed scars on the anterior neck, which were enhanced when the neck was hyperextended 1 month after the second treatment session with DCA for SMF reduction. The patient had no other symptoms, and the depressed scars were localized to the injection sites of DCA. After 2.5 years of follow-up, the dimpled scars were considered permanent as no improvement was observed over time.^40^

The third case was that of a 44-year-old female with edema, pain, and erythema that progressed to a deep ulcerative wound 1 week after DCA injections for SMF reduction. There was no evidence of abnormal fluid collection; however, cultures confirmed the presence of *Pseudomonas aeruginosa*. The patient did not respond to antibiotic therapy, local debridement, and hyperbaric oxygenation. Pathological examination revealed a severe skin reaction with neutrophilic infiltrates, compatible with the diagnosis of neutrophilic dermatosis. The patient was treated with methylprednisolone, topical tacrolimus, and infliximab.^41^

Considering these cases, the authors believe that skin ulceration and necrosis are direct consequences of intradermal injections; therefore, it is imperative to appreciate the tactile feedback and resistance perceived from the dermis. Intradermal injections have a higher resistance and show a visible intradermal wheel, and repeated intradermal injections cause blebbing on the skin surface (Figure 3).

*Abscess*: A single case of abscess after DCA treatment for SMF reduction has been reported in the literature. The patient was a 44-year-old female who presented with a submental abscess 2 days after the treatment session. The swelling, erythema, and pain in the submental region were followed by thickening and subcutaneous stranding in the submental region, inflammatory changes within the fat tissue, and reactive lymphadenopathy. The patient did not respond to broad-spectrum intravenous antibiotic therapy. Thereafter, surgical drainage and debridement were performed, followed by the administration of intravenous antibiotics for 2 weeks to resolve the complication.^42^

The risk of abscess formation can be decreased by employing good aseptic techniques and avoiding injection through the skin that is compromised secondary to any active infection, acne pustules, or folliculitis. The treatment of skin and soft-tissue infections usually requires the administration of systemic oral antibiotics. A true abscess may require incision and drainage of the infection.

*Intraarterial injection*: To date, 2 case reports documenting the inadvertent intraarterial injection of DCA have been reported in the literature. The first case was that of a 42-year-old female who was treated with ATX-101 for SMF reduction. When ATX-101 was injected after a reflux maneuver 1 cm below the mandible to the right of the midline, the patient immediately complained of severe lower gum and tooth pain. The pain was rated as 10/10 on visual analog scale, and the injections were stopped. Immediate treatment involved ineffective attempts at aspirating blood or any DCA that had been injected. Thereafter, the patient became diaphoretic and mildly hypotensive (with a feeling of fainting), as she experienced severe right jaw pain and right-sided headache. She was placed in the Trendelenburg position, and an attempt was made to recannulate the artery. The extravasation protocol for sclerosing agents was followed, and the area was flushed with hyaluronidase (200 units in 5 cc of normal saline), as it disrupts the interstitial barrier to increase the distribution and absorption of the extravasated drug. After referring to the protocol for intraarterial injection of sclerosing agents, prednisolone (30 mg), and aspirin (325 mg) were administered. Local treatment involved massaging the area and applying warm compresses. The patient was administered diazepam (5 mg) for severe anxiety. A reticular pattern was observed on the submental region, chin, and lower gums, with the absence of capillary refill, after the application of digital pressure. Owing to the lack of improvement after 30 min, the patient was transferred to a hyperbaric oxygen center within 90 min of the event. The 2 h hyperbaric treatment dramatically improved the reticular pattern, with a significant decrease in pain. Eight hours after the injection, an increase in submental and submucosal ecchymoses was noted. After 24 h of the event, the patient complained of sore throat, along with moderate swelling of the tongue, gums, and submucosal region. The patient underwent additional hyperbaric treatment sessions at 48 and 72 h after the event. An 8 mm eschar developed at the injection site, accompanied with swelling that recurred each morning for 1 week. The eschar was kept moist, and both aspirin (325 mg/day) and prednisone (30 mg/day; tapered over 10 days) were continued, which significantly decreased the swelling. Dental consultation for tooth pain showed that dentition was not compromised. The residual reticulated erythematous area and eschar were treated with pulsed dye laser treatment weekly for 3 weeks and monthly for 4 months. After 6 months, a small residual textural area, which was not cosmetically noticeable, remained. In addition, the authors described a treatment algorithm that included the infusion of heparin and prostaglandin E1 along with hyperbaric oxygen.^43^

The second report was a case of DCA injection into the facial artery that caused skin necrosis. Immediately after injecting DCA into the right submental region, purpura with blanching of the skin was observed inferolateral to the antegonial notch, extending 2 cm along the path of the facial artery. Immediate treatment involved the injection of normal saline into the facial artery and surrounding skin to dilute the extravasation of surfactants, similar to that done for sclerosing agents. Although the patient did not report immediate pain, he complained of soreness throughout the entire treatment area later. The tenderness below the right jawline further increased the following day. The patient was lost to follow-up and returned on Day 5 with a tender, reticulated, erythematous patch, along with a dusky violaceous hue in the center on the right mandible. Thereafter, the patient was initiated on 325 mg aspirin daily and was advised to apply a bland dimethicone-based ointment to keep the area moist. Twelve days after the treatment session, 2 shallow ulcers (0.5-1 cm) were observed covering the treated area. The ulcers were treated with a hydrocolloid dressing every 3 days until full re-epithelialization occurred. Four weeks after the treatment session, a 1.5 cm, slightly indurated erythematous linear plaque was observed along the mandible, which was treated with pulsed dye laser treatment (3 bimonthly sessions). The third pulsed dye laser treatment session was combined with a fractionated 1550 nm erbium-doped laser to target residual uneven skin texture and scarring. After 3 weeks, the patient underwent another pulsed dye laser treatment session, immediately followed by a fractionated CO_2_ laser therapy. The patient expressed satisfaction with the cosmetic outcome and did not return for follow-up.^44^

**Figure 4.**
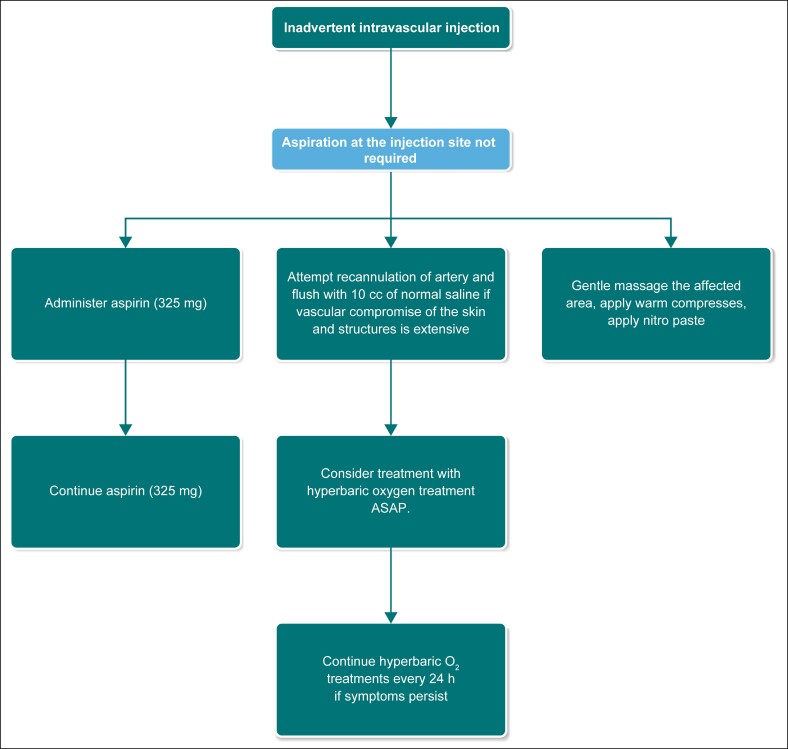
Treatment algorithm for intraarterial injection, modified from Lindgren and Welsh.^43^

The authors believe that it is not necessary to aspirate before each injection of ATX-101, and even in the case of an inadvertent intraarterial injection, we do not consider it necessary to aspirate. To prevent intraarterial injection, an understanding of both the vascular anatomy and the location of vascular structures is crucial. Based on the serious complication described above, it can be inferred that patients’ vital signs, hemodynamic status, and condition of the region supplied by the artery determine the severity of the complication (Figure 4).

*Incorrect product injection and accidental injection into incorrect areas (eg, lip and temple)*: The authors recommend handling only one product at a time based on the specific area being treated, to avoid confusion and potential mishandling of toxins, fillers, ATX-101, and any other syringes with medical devices/drugs.

The best practice is to label syringes clearly, such that the correct syringe with the correct product is confirmed before administering the injection to the patient. Treatment should be based on the AE associated with the specific area being treated by the respective product.

*Pyoderma gangrenosum*: A case of pyoderma gangrenosum has been previously reported. Pyoderma gangrenosum is a serious ulcerating skin disease that mostly requires empirical treatment. A correct diagnosis can be achieved by observing the ulcer edge, which is often undermined (worn and damaged), along with erythema and induration of the surrounding skin. The ulcer typically exhibits a “cat's paw” appearance, as it often starts as a small papule or the collection of papules that break down to form small ulcers, coalescing to form a single ulcer from the central area of necrosis.^45^

It is imperative to understand the inflammatory nature of pyoderma gangrenosum, as the treatment guided by the disease presentation during the physical examination may be directed towards an infective etiology. Erroneous diagnosis and management by performing an incision and drainage may lead to further tissue damage and instigation/flaring of pyoderma gangrenosum (Figure 3).

*Ulnar nerve palsy*: Injections administered near the sensory and motor nerves can lead to deficits consistent with a demyelination type injury. In 1 case, an injection near the medial epicondyle of the arm led to motor deficits in the hand, indicative of ulnar nerve palsy. The clinical manifestation was temporary and completely resolved within ∼1 month, with no residual motor defects.

## DISCUSSION

The focus of this review is to provide recommendations for the best practice approaches and insights into the implications of using adipocytolytic agents in aesthetic medicine. Overall, it is evident that adherence to a standard treatment protocol with appropriate foresight can help prevent the occurrence of SAEs. Owing to the absence of a treatment protocol for the management of SAEs associated with the intravascular injection of ATX-101, an overzealous protocol for sclerosing agents appears to have been followed in the published literature^43^ and is likely to be applied by anxious surgeons in the real world. However, the authors recommend that an appropriate assessment of the seriousness should be made based on the patients’ vital signs, hemodynamic status, and condition of the region supplied by the artery.

In addition to the standard limitations that apply to the use of injectable nonspecific cytolytic agents for aesthetic medicine, including the need for a skilled professional with an understanding of aesthetic requirements, this procedure requires multiple injections and treatment sessions.

## CONCLUSIONS

In addition to standard procedures for ATX-101 administration, the authors recommend investigating other potential causes of SMF accumulation and jowling mechanism. Although surgery is preferable for jowling caused by compartment displacement (ptosis), fat-reducing treatments, such as ATX-101, are contraindicated for jowling caused by subcutaneous tissue atrophy. Overall, proactive measures that can be employed to prevent AEs include avoiding intradermal injections to prevent skin ulceration/necrosis, injecting lidocaine to check for smile asymmetry as an indication of MMN proximity, administering 1 to 2 mm deeper injections in males to prevent alopecia, employing good aseptic techniques to prevent abscess formation, injecting 1 product at a time using correctly labeled syringes, and confirming the diagnosis of pyoderma gangrenosum before treating it as an infection.
